# Methanol intoxication and acute kidney injury: pathophysiological mechanisms and therapeutic approaches

**DOI:** 10.1590/2175-8239-JBN-2025-0344en

**Published:** 2026-05-22

**Authors:** Guilherme Nobre Nogueira, Camilla Beatriz Marinho Teles, Marcos Vinicius Sousa Varão, Anderson Carneiro Costa, Gabriel Moreira de Lima Ramos, Elizabeth De Francesco Daher

**Affiliations:** 1 Universidade Federal do Ceará, Faculdade de Medicina, Departamento de Medicina Clínica, Fortaleza, CE, Brazil.; 2 Universidade Federal do Ceará, Faculdade de Medicina, Departamento de Medicina Clínica, Programa de Pós-Graduação em Ciências Médicas, Fortaleza, CE, Brazil.

**Keywords:** Acute Kidney Injury, Fomepizole, Formic acid, Renal Dialysis, Methanol, Mitochondrial Diseases, Toxicity, Kidney Diseases.

## Abstract

Methanol intoxication is a potentially lethal condition that primarily affects the central nervous system, but it can also induce significant renal damage. Acute kidney injury (AKI) in this context is often underestimated, despite being an important prognostic marker associated with increased mortality and morbidity. This review aims to elucidate the pathophysiological mechanisms linking methanol intoxication to AKI, describe the cellular and metabolic pathways involved, and discuss current therapeutic approaches for renal protection and recovery. A descriptive and analytical review was conducted through searches in the PubMed, Embase, and Cochrane Library databases, including studies published between 2000 and 2025. Eligible articles addressed methanol-related nephrotoxicity, AKI mechanisms, or treatment strategies involving fomepizole, hemodialysis, and renal support. The nephrotoxic effects of methanol are mediated by the accumulation of formic acid, which inhibits mitochondrial cytochrome oxidase, leading to tissue hypoxia, oxidative stress, and cellular apoptosis. The main renal alterations include osmotic nephrosis and acute tubular necrosis, frequently associated with metabolic acidosis, rhabdomyolysis, or hemolysis. Risk factors such as anemia, sepsis, volume depletion, and acute pancreatitis exacerbate renal injury. AKI is linked to higher rates of multiple organ failure and in-hospital mortality. Methanol-induced AKI results from multifactorial mechanisms involving mitochondrial dysfunction, oxidative stress, and hemodynamic instability.

## INTRODUCTION

Methanol (CH^3^OH) is an industrial alcohol widely used in laboratories, household products, antifreeze, windshield washer fluids, solvents, and as a precursor for plastic production. Due to its low cost, it is frequently used in the manufacture of adulterated or illegal alcoholic beverages^1^. This substance is toxic due to its rapid intestinal absorption and subsequent metabolism in the body. It is metabolized by alcohol dehydrogenase, being converted into an aldehyde (formaldehyde), and subsequently oxidized into formic acid by aldehyde dehydrogenase^2^.

This final metabolite is primarily responsible for systemic toxicity, causing severe metabolic acidosis and characteristic lesions in the central nervous system. The underlying mechanism involves the inhi­bition of cytochrome c oxidase in the mitochondria by formic acid, compromising cellular function and leading to tissue hypoxia^1,3^.

Although the kidney is not traditionally considered a primary target organ in methanol intoxication, acute kidney injury (AKI) is a frequent and potentially severe complication in patients intoxicated by this alcohol. The pathophysiology of methanol-associated AKI is multifactorial and complex. Three main pathophysiological mechanisms have been proposed: (1) indirect factors, such as rhabdomyolysis (with myoglobinuria) and hemolysis (with hemoglobinuria), whose toxicity is intensified by metabolic acidosis, which lowers urinary pH and favors precipitation and intratubular cast formation; (2) renal hypoperfusion, resulting from hemodynamic instability, shock, and volume depletion, contributing to ischemic injury; and (3) a direct toxic effect of formic acid, which, associated with osmotic changes, may preferentially affect proximal tubular cells, leading to hydropic changes and tubular dysfunction^3^.

AKI in patients with methanol intoxication is a marker of severity and is associated with a significant increase in hospital mortality, higher morbidity, and greater utilization of hospital resources, including Intensive Care Unit (ICU) beds, mechanical ventilation, and renal replacement therapy^2,3^.

Furthermore, this study aims to identify the potential pathophysiological processes of AKI associated with methanol intoxication, describing the nephrotoxic mechanisms of the substance. Thus, this work aims to address the treatment and support of patients with methanol intoxication, focusing on the management of kidney injury patients.

## METHODS

This work is a descriptive and analytical review aimed at collecting and critically analyzing the available evidence on the pathophysiological mechanisms of methanol intoxication, its relationship with AKI, and current therapeutic strategies. The review format was selected due to the scarcity of randomized con­trolled trials and the predominance of observational studies and case reports, which limit the feasibility of a quantitative meta-analysis.

The article aimed to answer the following ques­tion: “What are the pathophysiological mecha­nisms that explain the relationship between methanol intoxication and the development of acute kidney injury, and what are the most effective therapeutic approaches for treating this condition?”.

The search strategy employed relevant terms across the PubMed, Embase, and Cochrane Library databases: (“Methanol” OR “Methyl Alcohol” OR “Methanol Poisoning” OR “Methanol Intoxication” OR “Methanol Toxicity” OR “Methanol Exposure” OR “Methanol-Induced Injury” OR “Toxic Alcohols” OR “Alcohol Poisoning” OR “Alcohol Intoxication”) AND (“Acute Kidney Injury” OR “AKI” OR “Renal Failure” OR “Acute Renal Failure” OR “Acute Renal Injury” OR “Renal Dysfunction” OR “Kidney Failure” OR “Kidney Injury” OR “Kidney Damage” OR “Renal Impairment” OR “Nephrotoxicity” OR “Toxic Nephropathy”) AND (“Pathophysiology” OR “Mechanism of Injury” OR “Mechanism of Toxicity” OR “Mechanism of Action” OR “Pathogenesis” OR “Molecular Mechanism” OR “Mitochondrial Dysfunction” OR “Oxidative Stress” OR “Cellular Toxicity” OR “Metabolic Acidosis”) AND (“Treatment” OR “Therapy” OR “Management” OR “Clinical Management” OR “Supportive Care” OR “Detoxification” OR “Antidote”).

This review included original research articles, population-based studies, case reports, and case series that addressed the pathophysiological mechanisms of methanol intoxication, its association with AKI, the clinical implications or the therapeutic management involving antidotes, dialysis, or renal support. Eligible studies were full-text open-access publications in English, Portuguese, or Spanish; peer-reviewed; and published between January 2000 and October 2025. Studies were excluded if they lacked full-text availability, focused exclusively on ethylene glycol or other alcohols without mentioning methanol, presented insufficient renal or therapeutic data, were duplicates, or were reviews or studies with poor methodological quality.

Study screening was conducted in two stages: initial evaluation of titles and abstracts to exclude irrelevant studies, followed by a full-text review to confirm eligibility. Two independent reviewers per­formed the selection process, and any disagreements were resolved by consensus. Data extraction was standardized and included information on authors, publication year, study type, population/sample characteristics, pathophysiological mechanisms, type and severity of AKI, therapeutic strategies (fomepizole, ethanol, hemodialysis, folinic acid, renal support), and renal or clinical outcomes.

Due to the narrative design of this review, data synthesis was qualitative and organized into two thematic axes: (1) the pathophysiology of methanol intoxication leading to AKI, including metabolism, mitochondrial toxicity, and metabolic acidosis, and (2) therapeutic approaches and renal support, encompassing antidotes, dialysis, and metabolic correction. A total of 574 articles were initially identified; after removing 36 duplicates, 538 were screened based on titles and abstracts, resulting in 17 full-text articles included in the final analysis.

## RESULTS

### Pathophysiology of Methanol Intoxication and Mechanisms of Nephrotoxicity

The pathogenesis of AKI in methanol intoxication involves several interacting mechanisms, acting both systemically and locally^4^. To fully understand its renal implications, it is essential, first and foremost, to examine the metabolism and pathophysiology of methanol intoxication.

Methanol is rapidly absorbed through the gastrointestinal tract, with an average half-life of five minutes and peak absorption occurring within thirty to sixty minutes after ingestion^1,5,6^. The initial and most critical metabolic step is the oxidation of methanol to formaldehyde, a reaction catalyzed by the hepatic enzyme alcohol dehydrogenase (ADH)^3,6,7,8^. Formaldehyde, a highly reactive intermediate, has an extremely short half-life of one to two minutes before it is rapidly converted by aldehyde dehydrogenase (ALDH) into formic acid (or formate)^3,6,7,8^. Subsequently, formic acid dissociates into hydrogen ions and the formate anion, the latter being metabolized by the enzyme 10-formyltetrahydrofolate dehydrogenase. This enzyme catalyzes the combination of formate with tetrahydrofolate to form 10-formyltetrahydrofolate, which is ultimately degraded to carbon dioxide and water^5,8^. Tetrahydrofolate derives from folic acid, and its concentration depends on adequate dietary folate intake and efficient regeneration during formate oxidation, a process often limited or saturated in humans^5,8^. Consequently, the half-life of formic acid can extend up to twenty hours, resulting in sustained accumulation of formate in the bloodstream and a strong correlation between serum formate levels and methanol toxicity^3,5^. It has also been proposed that the slow clearance of formic acid may reflect zero-order kinetics affecting both methanol and formic acid metabolism, potentially compounded by continuous recycling of formic acid and protons with chloride ions in the kidney^5^.

The accumulation of formic acid constitutes the principal mechanism underlying the profound meta­bolic acidosis observed in methanol intoxication^8^. Formic acid inhibits cytochrome c oxidase, thereby blocking oxidative phosphorylation within mitochondria—an essential process for cellular respiration—and inducing tissue hypoxia^5,7,8^. The resulting mitochondrial dysfunction triggers a shift toward anaerobic metabolism, leading to excessive lactate production and worsening of the metabolic acidosis initially caused by formic acid dissociation^3,5,7^.

The combined accumulation of formic acid and lactic acid, arising from impaired cellular oxygen utilization, produces a markedly elevated anion gap. The ensuing severe acidosis further aggravates the condition by promoting the intracellular diffusion of formic acid^3,5,7^. Compared with formate, undissociated formic acid is approximately three times more potent as an inhibitor of mitochondrial cytochrome oxidase, the terminal enzyme in the mitochondrial electron transport chain^5^. Consequently, tissues with high metabolic demand, limited energy reserves, and abundant mitochondria, such as the optic nerve and retina, are particularly vulnerable to this hypoxic injury, leading to the characteristic visual disturbances of methanol poisoning^1,7,8^.

The kidney injury induced by toxic metabolites, including formic acid, is mediated by universal downstream pathways common to many forms of AKI. One of these mechanisms is oxidative stress, resulting from excessive production of reactive oxygen species (ROS) secondary to mitochondrial dysfunction. The kidney is particularly susceptible to ROS-induced damage, especially proximal tubular cells. This ROS overproduction overwhelms the intrinsic antioxidant defense systems, leading to depletion of reduced glutathione (GSH) and accumulation of lipid peroxidation end-products (MDA) and protein oxidation markers (AOPP, PC)^9,10,11^. Moreover, mitochondria play a central role in regulating cell death processes, including apoptosis, necrosis, and autophagy, by triggering programmed cell death pathways^12^. In addition, there is intense activation of inflammatory signaling pathways, particularly the nuclear factor kappa B (NF-κB) pathway. Markers of cellular stress and inflammation, such as kidney injury molecule-1 (KIM-1), become highly expressed following tubular injury^9,12^.

Additionally, methanol ingestion often produces a marked osmolal gap (OG), with a serum methanol concentration of 50 mg/dL corresponding to an approximate increase of 17 mOsm/kg H^2^O^5^. Given this, methanol intoxication has been associated with a specific morphological change in the proximal tubules resembling osmotic nephrosis. This histopathological pattern is marked by pinocytosis and lysosomal accumulation of sodium and water, resulting in cellular swelling and vacuolar distention of proximal tubular cells, without evidence of glomerular involvement^4^, as described in two renal biopsies reported by Verhelst^3^. Calcium influx into tubular cells has been proposed as one of the possible mechanisms of toxicity. Furthermore, sodium chloride absorption in the proximal tubule is markedly stimulated by formate, which may account for the hydropic changes observed in proximal tubular epithelial cells in these biopsies, ultimately leading to acute tubular necrosis (ATN)^3,13^. Osmotic nephrosis is often a reversible form of early proximal tubular injury, with functional recovery typically occurring after withdrawal of the causative agent, although rare cases of persistent renal failure have been documented^4^.

**Figure 1:**
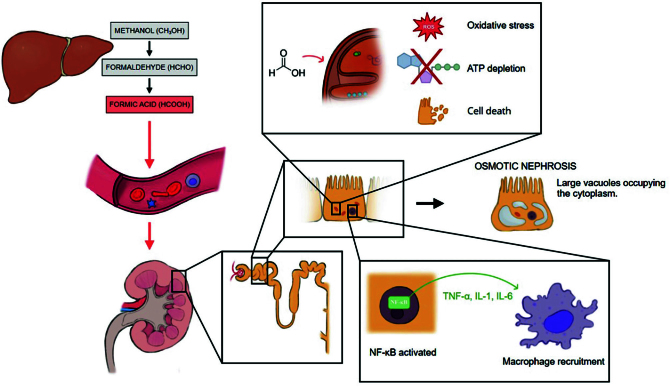
Pathophysiology of methanol intoxication leading to nephrotoxicity.

In some cases, the kidney is not considered a direct target of methanol toxicity; rather, AKI is viewed as a complication secondary to disease progression, resulting from indirect mechanisms of injury^3^. Hemolysis is considered one of these possible mechanisms. It has been suggested that formic acid may induce hemolysis through a cytotoxic effect on erythrocytes—a process more closely related to the degree of metabolic acidosis than to a direct hemolytic action on red blood cells^3^. Heme proteins can promote kidney injury through three main mechanisms: reduction of renal perfusion, direct cytotoxicity, and the formation of pigmented casts leading to tubular obstruction^1^. Rhabdomyolysis is not a common complication of methanol intoxication; however, it has also been identified as an indirect risk factor for AKI^1^. The mechanism involves the precipitation of myoglobin and hemoglobin within the renal tubules, leading to tubular obstruction. The precipitation of these pigments is exacerbated by the acidic urine pH^6,7^. In one of the studies analyzed, 7 out of 11 cases of AKI presented with myoglobinuria and hemoglobinuria^3^. Elevated serum creatine phosphokinase (CPK) levels have been reported in epidemic outbreaks of methanol intoxication, suggesting an association with rhabdomyolysis^13^. However, no direct toxic effect of methanol on skeletal muscle has been clearly demonstrated in the literature, nor have any direct hemolytic properties been attributed to it^3^. Finally, acute pancreatitis, a common complication of severe poisoning, can contribute to AKI through the systemic release of inflammatory mediators and pancreatic enzymes from necrotic tissue, resulting in direct nephrotoxicity^7^, as shown in Figure 1.

Abbreviations – ATP: Adenosine triphosphate; NF-κB: Nuclear factor kappa B; TNF-α: Tumor necrosis factor alpha; IL-1: Interleukin 1; IL-6: Interleukin 6.

Notes – Methanol is metabolized to formic acid, causing mitochondrial dysfunction, oxidative stress, ATP depletion, and cell death. In the kidney, proximal tubular injury results in osmotic nephrosis with cytoplasmic vacuolization, NF-κB activation, release of pro-inflammatory cytokines, macrophage recruitment, and progression to acute tubular necrosis.j

### Therapeutic Approaches

The management framework for methanol poisoning, encompassing the critical approach to associated AKI, rests upon several fundamental interventions: halting the production of toxic metabolites, facilitating the elimination of both methanol and formic acid, correcting life-threatening metabolic acidosis, and administering comprehensive supportive care^8^.

### Ethanol as an ADH Inhibitor

Ethanol may be used as an alternative antidote when fomepizole is unavailable, acting as a competitive substrate for alcohol dehydrogenase (ADH), with an affinity approximately 10–20 times higher than that of methanol. The therapeutic goal is to maintain a serum ethanol concentration of approximately 100 mg/dL (22 mmol/L), which is sufficient to saturate ADH and prevent the metabolism of methanol into formaldehyde and formic acid^7,8,12,14^.

Intravenous ethanol is typically administered as a 10% ethanol solution. A loading dose of 0.6–0.8 g/kg is given over 30–60 minutes, followed by a maintenance infusion of 66–154 mg/kg/h, adjusted according to age, chronic alcohol use, and serum ethanol levels. During hemodialysis, ethanol clearance is markedly increased, and the infusion rate should be increased by two- to threefold to maintain therapeutic serum concentrations^7,8,12,14^.

When intravenous ethanol is unavailable, oral or nasogastric administration may be used. A loading dose equivalent to 0.8 g/kg is administered, followed by 0.1–0.2 g/kg/h, using diluted ethanol (e.g., spirits diluted to a known concentration). Frequent monitoring of serum ethanol levels is required to ensure adequate ADH inhibition and to avoid toxicity^7,8,12,14^.

### Inhibiting Methanol Metabolism: ADH Blockade

Preventing the conversion of methanol to formaldehyde and subsequently to formic acid is a primary therapeutic goal. This is accomplished by inhibiting ADH, the rate-limiting enzyme in this pathway^8^. The two available ADH inhibitors are fomepizole and ethanol.

**Fomepizole (4-methylpyrazole):** Fomepizole exhibits a markedly higher affinity for ADH (estimated to be 500 to 1000 times greater than that of ethanol), enabling complete enzyme inhibition at relatively low serum concentrations (target >0.8 mg/L or 10 µmol/L)^8^. Clinical evidence from observational studies indicates its effectiveness in reducing formic acid levels, improving metabolic acidosis, and potentially enhancing visual outcomes in methanol poisoning^8,15^. Its advantages include a favorable side-effect profile (headache, nausea, and dizziness reported), more predictable pharmacokinetics with reduced need for intensive blood level monitoring, absence of sedative effects (unlike ethanol), and no contribution to the serum osmolal gap, which can be useful for indirectly tracking methanol elimination if direct levels are unavailable^8,15^. Consequently, fomepizole is the preferred agent when accessible. Significant limitations are its substantial cost and variable availability across healthcare facilities. As fomepizole is removed by hemodialysis, dose adjustments (increasing the frequency or adding supplemental doses) are necessary during renal replacement therapy. A standard regimen involves an intravenous loading dose of 15 mg/kg, followed by scheduled maintenance doses (e.g., 10 mg/kg every 12 hours for 4 doses, then 15 mg/kg every 12 hours), with increased frequency during dialysis^8^. Fomepizole (also known as 4-MP) additionally inhibits CYP2E1, an enzyme involved in acetaminophen metabolism, which has been shown to protect against acetaminophen-induced AKI in mice^16^, although ADH inhibition remains the primary therapeutic mechanism cited for methanol poisoning.

**Ethanol:** Ethanol acts as a competitive substrate for ADH, possessing an affinity approximately 10–20 times higher than that of methanol^8^. Achieving and maintaining a serum ethanol concentration around 100 mg/dL is required for effective enzyme saturation and inhibition of methanol metabolism^7,8^. Ethanol’s primary advantages are its low cost and universal availability. However, it is associated with significant disadvantages, including central nervous system depression (complicating neurological assessment), the need for frequent blood level monitoring and dose adjustments (typically necessitating ICU admission), elevation of serum osmolality (obscuring the osmolal gap as a monitoring tool), risk of hypoglycemia, potential hepatic effects. Furthermore, ethanol is substantially removed by hemodialysis, requiring significantly increased infusion rates during the procedure. Despite these drawbacks, ethanol remains a viable alternative when fomepizole cannot be used. Dosing involves a loading dose followed by a maintenance infusion, which must be substantially increased during hemodialysis^8^.

### Indications for ADH Blockade

Guideline criteria for initiating ADH blockade (either fomepizole or ethanol), as cited by Kraut and Kurtz (2008), include confirmed plasma methanol levels >20 mg/dL; a documented recent ingestion history coupled with an osmolal gap >10 mOsm/L; or strong clinical suspicion based on presentation (e.g., visual symptoms, acidosis) combined with supportive laboratory findings (arterial pH <7.3, serum bicarbonate <20 mEq/L, or osmolal gap >20 mOsm/L). Early administration is critical for efficacy while unmetabolized methanol remains present^8,15^.

### Extracorporeal Removal: Hemodialysis

**Figure 2:**
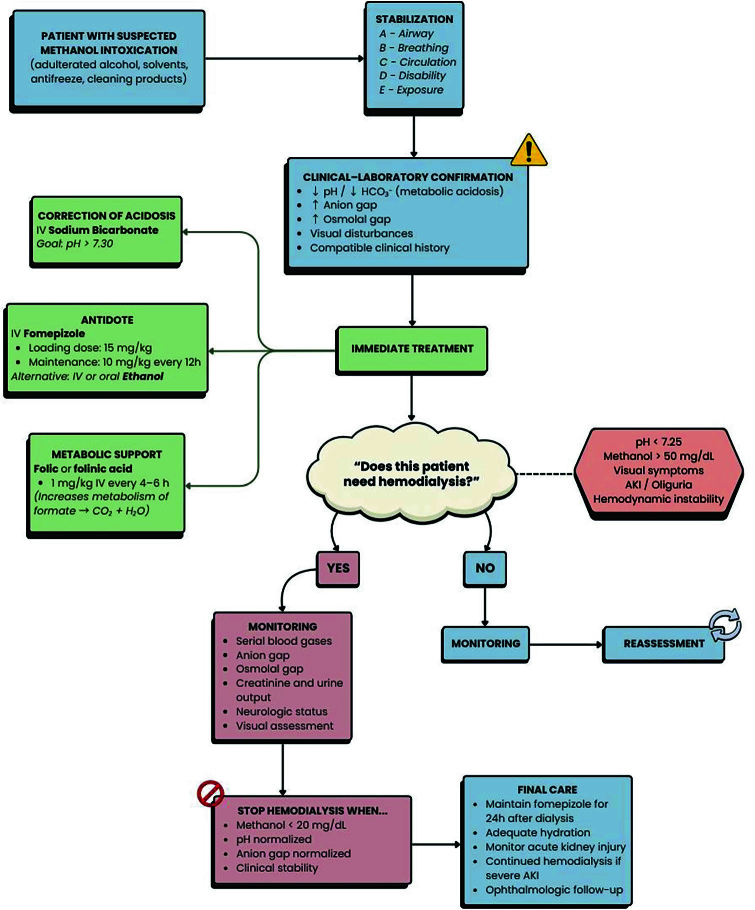
Diagnostic and therapeutic flowchart for methanol intoxication, including indications for hemodialysis.

Hemodialysis (HD) is a crucial intervention in severe methanol poisoning, primarily aimed at accelerating the removal of both methanol and its toxic metabolite, formic acid, from circulation^7,8^, as shown in Figure 2. The physicochemical properties of these substances (low molecular weight, minimal protein binding, and low volume of distribution) make them highly amenable to removal by dialysis^8,17^. Intermittent hemodialysis provides the most rapid clearance compared with other renal replacement modalities such as continuous therapies or peritoneal dialysis^8^.

#### Indications for hemodialysis

The decision to initiate HD is based on clinical and laboratory parameters indicative of severe poisoning. Commonly accepted indications include:

- **Elevated serum methanol levels:** Thresholds vary, often cited as >50 mg/dL or sometimes lower, such as >25 mg/dL^7,8^.
- **Severe metabolic acidosis:** Defined by parameters such as arterial pH < 7.1, < 7.25, or < 7.30, frequently refractory to bicarbonate therapy alone^7,8^. Low serum bicarbonate, in itself, is a strong predictor of mortality and may implicitly support the indication for dialysis^7^.
- **Visual impairment:** Any degree of visual disturbance attributed to methanol is often considered an indication^7,8^. Blindness at presentation is associated with a significantly increased risk of mortality^7^.
- **Presence of AKI / renal failure:** Kidney injury alone constitutes an indication for renal replacement therapy, particularly when severe or when contributing to acidosis/electrolyte disturbances^7,8^.
- **Clinical deterioration:** Including worsening vital signs (e.g., persistent hypotension) or significant electrolyte disturbances (such as severe hyperkalemia, although typically less common than acidosis) not manageable by other means^7,8^.

While the primary role of HD is methanol removal, it also effectively eliminates formate. Studies have demonstrated that HD can substantially reduce the half-life of formate, especially in severe cases where endogenous clearance may be overwhelmed. Additionally, HD efficiently corrects metabolic acidosis through bicarbonate transfer from the dialysate^8^.

#### Timing and duration

Prompt initiation of HD, ideally concurrent with ADH blockade (fomepizole preferred), is recommended for most patients with significant methanol poisoning. This approach aims to expedite toxin clearance, manage acidosis, improve outcomes, and potentially mitigate the severity or development of AKI^8,15^. In practice, initiation within 3–5 hours of emergency department (ED) arrival has been achieved in outbreak settings^15^. The duration of HD is guided by the goal of reducing methanol levels to non-toxic concentrations (e.g., <16 mg/dL or <5 mmol/L, or undetectable) and resolving the metabolic acidosis (pH > 7.3). Kinetic formulas incorporating patient parameters and dialyzer characteristics can aid in estimating the required dialysis vintage^8^. Patients may require multiple HD sessions (one to three or more) depending on the initial methanol load, ongoing metabolism if ADH blockade is incomplete, and severity of poisoning^15^. Serial monitoring of methanol levels and acid-base status is essential to determine the appropriate endpoint of therapy.

### Correcting Metabolic Acidosis: Sodium Bicarbonate

Intravenous administration of sodium bicarbonate is a vital supportive measure to counteract the severe metabolic acidosis caused by formic acid accumulation^7,8,15^. Amelioration of acidemia can improve cardiovascular function (by enhancing myocardial contractility and vascular responsiveness to catecholamines). Theoretically, it may also reduce formic acid toxicity by decreasing its non-ionized fraction, thereby limiting tissue penetration (especially into the CNS and optic nerve) and possibly increasing its urinary excretion via ion trapping. Bicarbonate therapy is generally indicated when arterial pH falls below 7.3^7,8^, with more aggressive correction often warranted for severe acidemia (pH < 7.1–7.2)^8^. An initial bolus dose of 1–2 mEq/kg may be administered, followed by continuous infusion or intermittent boluses, titrated based on frequent arterial blood gas monitoring to achieve a target pH (e.g., >7.3)^7^. It is crucial to avoid overcorrection and to monitor for potential complications, such as hypokalemia or hypocalcemia. Hemodialysis remains the most effective method for correcting severe acidosis due to its capacity to deliver large amounts of bicarbonate buffer^8^.

### Supportive Management

Intensive supportive care tailored to the patient’s clinical status is crucial alongside specific treatments. Airway management, including endotracheal intubation and mechanical ventilation, is frequently required in patients presenting with severely depressed consciousness (low Glasgow Coma Scale scores), seizures, or respiratory compromise due to severe acidosis or CNS depression^7,15^. Cardiovascular support is critical. Hypotension, a marker of severe toxicity and poor prognosis, requires aggressive management with intravenous fluid resuscitation and, often, vasopressor or inotropic support (e.g., norepinephrine) to maintain adequate mean arterial pressure (MAP) and organ perfusion, including renal perfusion^7,8^. Gastrointestinal decontamination methods, such as gastric lavage or activated charcoal, are generally considered ineffective unless performed within a very short window (30–60 minutes) after ingestion, due to the rapid absorption of methanol^8^. Continuous and vigilant monitoring of vital signs (heart rate, blood pressure, respiratory rate, temperature), neurological status (GCS), acid–base balance (serial arterial blood gases), electrolytes (especially potassium), glucose, renal function (creatinine, BUN, urine output), and visual acuity is mandatory throughout treatment^7,8^. Seizures, if they occur, should be managed with benzodiazepines according to standard protocols^15^.

## DISCUSSION

### Prognostic Considerations and Timeliness

The clinical outcome following methanol poisoning, including the development and severity of AKI, is critically dependent on the severity of metabolic acidosis at presentation and, crucially, the time interval between ingestion and the institution of effective therapies, such as ADH blockade and hemodialysis. Factors strongly predictive of mortality and significant morbidity (including AKI and permanent visual loss) include severe acidemia (pH < 7.1, markedly low bicarbonate, high anion gap, high lactate), profound coma (low GCS), hemodynamic instability (hypotension, low MAP), the presence of blindness upon admission, and evidence of AKI (elevated serum creatinine)^7,8^. Delays in seeking medical attention, influenced by factors such as lack of awareness or fear of legal repercussions in certain regions, significantly increase the risk of adverse outcomes, especially irreversible blindness^7^. Conversely, case series and outbreak reports suggest that rapid diagnosis coupled with timely initiation of combination therapy – ADH blockade (preferably with fomepizole) and early, effective hemodialysis – can result in substantially lower mortality rates and better functional recovery compared to scenarios involving delayed treatment or less aggressive management^15^. Therefore, mini­mizing the time-to-treatment interval is paramount to limiting formic acid accumulation and preventing or mitigating irreversible damage to the kidneys, eyes, and central nervous system^7,15^.

### Risk Factors For AKI and Prognosis

AKI is frequently exacerbated by systemic complications of severe poisoning, notably profound metabolic acidosis and hypotension, reflected by decreased mean arterial pressure (MAP)^7,14^. This hemodynamic instability compromises renal perfusion and enhances the toxic effects of formate. In addition, patients with pre-existing chronic kidney disease (CKD) or impaired renal function are at even greater risk for methanol-related nephrotoxicity, specifically the development of osmotic nephrosis, due to their reduced capacity to metabolize and excrete the toxic compound^4^.

In a cohort study by Thongprayoon et al. (2021) involving 603 hospitalized patients with a primary diagnosis of methanol poisoning, 135 individuals developed AKI at admission. Multivariate analysis identified anemia (OR 3.43; 95% CI 3.43–6.62, p < 0.001), hypertension (OR 1.86; 95% CI 1.10–3.15, p = 0.02), volume depletion (OR 3.46; 95% CI 1.70–7.06, p = 0.001), sepsis (OR 6.91; 95% CI 2.38–20.05, p < 0.001), rhabdomyolysis (OR 6.25; 95% CI 1.84–21.26, p = 0.003), and acute pancreatitis (OR 5.30; 95% CI 1.71–16.44, p = 0.004) as independent risk factors for AKI in patients with methanol intoxication^1^. Among these, sepsis showed the strongest association with AKI. This may be explained by renal hypoperfusion due to hemodynamic instability, microcirculatory dysfunction, and a systemic pro-inflammatory response leading to tubular injury—predominantly acute tubular necrosis—and subsequent mito­chondrial dysfunction, further aggravated by the intrinsic cytotoxicity of formic acid^1,7^.

Furthermore, it has also been demonstrated that patients with AKI had a significantly greater requirement for invasive mechanical ventilation (OR 6.93; 95% CI 4.08–11.70; p < 0.001) and extracorporeal therapies (OR 4.06; 95% CI 2.58–6.38; p < 0.001) compared with those without AKI. Moreover, AKI was significantly associated with an increased risk of respiratory (OR 7.53; 95% CI 4.43–12.80, p < 0.001), circulatory (OR 3.15; 95% CI 1.41–7.04, p = 0.005), neurological (OR 2.66; 95% CI 1.55–4.57, p < 0.001), and hematological (OR 2.90; 95% CI 1.25–6.77, p = 0.01) failure. Notably, the presence of AKI conferred a 6.39-fold higher risk of in-hospital mortality compared with patients without AKI (OR 6.39; 95% CI 2.92–13.96, p < 0.001). These findings may be explained by the systemic consequences of renal dysfunction. AKI leads to salt and water retention, accompanied by decreased myocardial contractility and cardiac arrhythmias— both triggered by electrolyte and acid-base disturbances—which, together, contribute to the development of hydrostatic pulmonary edema. Additionally, systemic inflammation and uremia can increase pulmonary vascular permeability, resulting in non-hydrostatic pulmonary edema, which represents another mechanism of respiratory failure^1^.

These mechanisms are consistent with the general model of poisoning-induced AKI, which emphasizes the convergence of oxidative stress, mitochondrial injury, and inflammation as central mediators of toxic renal damage across various etiologies. Methanol fits within this paradigm, leading to mitochondrial failure and activation of inflammatory signaling pathways, particularly via the NF-κB pathway and the overexpression of kidney injury markers such as KIM-1. The combination of mitochondrial dysfunction and inflammatory stress explains the severe acidosis, tubular apoptosis, and delayed renal recovery observed in many cases^9,17,18,19^.

From a comparative perspective, methanol-induced nephropathy shares certain pathogenic similarities with ethylene glycol intoxication, another cause of acute toxic kidney injury. However, ethylene glycol primarily causes crystal-induced tubular obstruction due to calcium oxalate deposition, whereas methanol toxicity predominantly leads to metabolic and mitochondrial dysfunction. Despite these mechanistic differences, both conditions frequently culminate in acute tubular necrosis and clinically significant renal dysfunction. In methanol poisoning, the absence of crystal deposition highlights a distinct pathophysiological profile driven by biochemical inhibition rather than mechanical obstruction, thereby reinforcing the central role of acute kidney injury as a marker of disease severity and a key determinant of therapeutic decision-making, particularly regarding the early indication of renal replacement therapy^4,19,20^.

Beyond direct tubular toxicity, several secondary factors further exacerbate renal injury and directly influence nephrological management. Hemodynamic instability, dehydration, and hypo­tension impair renal perfusion and promote ischemic tubular damage, often necessitating aggressive hemodynamic optimization and early renal support^1^. Additionally, rhabdomyolysis and hemolysis, although less frequent, may contribute to pigment-induced nephropathy through the release of myoglobin and hemoglobin, especially in acidic urinary conditions, thereby lowering the threshold for initiating renal replacement therapy^3,8^. Sepsis, identified as an independent risk factor, intensifies systemic inflammation and mitochondrial dysfunction, creating a self-perpetuating cycle of renal and multiorgan injury. In this context, the presence and severity of acute kidney injury emerge not merely as complications but also as pivotal determinants of prognosis and therapeutic strategy, underscoring the critical role of the nephrologist in guiding timely dialysis initiation and comprehensive renal support^1,20^.

## CONCLUSION

Methanol intoxication is a medical emergency characterized by complex systemic toxicity. Among its complications, acute kidney injury stands out as both a marker of disease severity and a key determinant of prognosis. Renal damage is multifactorial, involving direct tubular toxicity from formic acid, oxidative stress, mitochondrial dysfunction, and hemodynamic instability. Early intervention with fomepizole, folinic acid, and hemodialysis, together with meticulous supportive care, significantly reduces mortality and improves renal outcomes. A comprehensive understanding of the renal pathophysiology of methanol poisoning is essential to guide clinical decision-making and to support the development of novel therapeutic strategies aimed at renal protection and recovery.

## Data Availability

The dataset supporting the results of this study is not publicly available.
