# Supplementary material for: Methanol intoxication and acute kidney injury: pathophysiological mechanisms and therapeutic approaches
Source: J Bras Nefrol. 2026 May 22;48(3):e20250344. doi: 10.1590/2175-8239-JBN-2025-0344en (PMC13205139; doi:10.1590/2175-8239-JBN-2025-0344en)
Supplement: Translation

# Translation: Intoxicação por metanol e injúria renal aguda: mecanismos fisiopatológicos e abordagens terapêuticas

**DOI:** 10.1590/2175-8239-JBN-2025-0344pt

**Authors:** Guilherme Nobre Nogueira, Camilla Beatriz Marinho Teles, Marcos Vinicius Sousa Varão, Anderson Carneiro Costa, Gabriel Moreira de Lima Ramos, Elizabeth De Francesco Daher

**Affiliations:** 1 Universidade Federal do Ceará, Faculdade de Medicina, Departamento de Medicina Clínica, Fortaleza, CE, Brazil.; 2 Universidade Federal do Ceará, Faculdade de Medicina, Departamento de Medicina Clínica, Programa de Pós-Graduação em Ciências Médicas, Fortaleza, CE, Brazil.

**Keywords:** Injúria Renal Aguda, Fomepizol, Ácido fórmico, Diálise Renal, Metanol, Doenças Mitocondriais, Toxicidade, Nefropatias.

## Abstract

A intoxicação por metanol é uma condição potencialmente letal que afeta principalmente o sistema nervoso central, mas que também pode induzir danos renais significativos. A injúria renal aguda (IRA), nesse contexto, é frequentemente subestimada, apesar de ser um importante marcador prognóstico associado ao aumento da mortalidade e da morbidade. Esta revisão tem como objetivo elucidar os mecanismos fisiopatológicos que vinculam a intoxicação por metanol à IRA, descrever as vias celulares e metabólicas envolvidas e discutir as abordagens terapêuticas atuais para a proteção e a recuperação renal. Foi realizada uma revisão descritiva e analítica por meio de buscas nas bases de dados PubMed, Embase e Cochrane Library, incluindo estudos publicados entre 2000 e 2025. Os artigos elegíveis abordaram a nefrotoxicidade relacionada ao metanol, mecanismos de IRA ou estratégias de tratamento envolvendo fomepizol, hemodiálise e suporte renal. Os efeitos nefrotóxicos do metanol são mediados pelo acúmulo de ácido fórmico, que inibe a citocromo c oxidase mitocondrial, resultando em hipóxia tecidual, estresse oxidativo e apoptose celular. As principais alterações renais incluem nefrose osmótica e necrose tubular aguda, frequentemente associadas à acidose metabólica, rabdomiólise ou hemólise. Fatores de risco como anemia, sepse, depleção volêmica e pancreatite aguda exacerbam a injúria renal. A IRA está associada a taxas mais elevadas de falência de múltiplos órgãos e mortalidade hospitalar. A IRA induzida por metanol decorre de mecanismos multifatoriais, envolvendo disfunção mitocondrial, estresse oxidativo e instabilidade hemodinâmica.

## INTRODUÇÃO

O metanol (CH^3^OH) é um álcool industrial amplamente utilizado em laboratórios, produtos domésticos, anticongelantes, fluidos de limpeza de para-brisa, solventes e como precursor na produção de plásticos. Devido ao seu baixo custo, é frequentemente utilizado na fabricação de bebidas alcoólicas adulteradas ou ilegais^1^. Essa substância é tóxica devido à sua rápida absorção intestinal e ao processo metabólico no organismo. Seu metabolismo é catalisado pela álcool desidrogenase, que o converte em um aldeído (formaldeído), sendo posteriormente oxidado a ácido fórmico pela aldeído desidrogenase^2^.

Esse metabólito final é o principal responsável pela toxicidade sistêmica, causando acidose metabólica grave e lesões características no sistema nervoso central. O mecanismo subjacente envolve a inibição da citocromo c oxidase nas mitocôndrias pelo ácido fórmico, comprometendo a função celular e levando à hipóxia tecidual^1,3^.

Embora os rins não sejam tradicionalmente considerados um órgão-alvo primário na intoxicação por metanol, a injúria renal aguda (IRA) é uma complicação frequente e potencialmente grave em pacientes intoxicados por esse álcool. A fisiopatologia da IRA associada ao metanol é multifatorial e complexa. Três principais mecanismos fisiopatológicos foram propostos: (1) fatores indiretos, como rabdomiólise (com mioglobinúria) e hemólise (com hemoglobinúria), cuja toxicidade é intensificada pela acidose metabólica, que reduz o pH urinário e favorece a precipitação e a formação de cilindros intratubulares; (2) hipoperfusão renal, resultante de instabilidade hemodinâmica, choque e depleção volêmica, contribuindo para injúria isquêmica; e (3) efeito tóxico direto do ácido fórmico, que, associado a alterações osmóticas, pode afetar preferencialmente as células dos túbulos proximais, levando a alterações hidrópicas e disfunção tubular^3^.

A IRA em pacientes com intoxicação por metanol é um marcador de gravidade e está associada a um aumento significativo da mortalidade hospitalar, maior morbidade e maior utilização de recursos hospitalares, incluindo leitos de Unidade de Terapia Intensiva (UTI), ventilação mecânica e terapia renal substitutiva^2,3^.

Este estudo busca identificar os possíveis processos fisiopatológicos da IRA associada à intoxicação por metanol, descrevendo os mecanismos nefrotóxicos da substância. Assim, propõe-se a abordar o tratamento e o suporte ao paciente intoxicado, com foco no manejo de pacientes com injúria renal.

## MÉTODO

Este trabalho é uma revisão descritiva e analítica destinada à coleta e à análise crítica das evidências disponíveis sobre os mecanismos fisiopatológicos da intoxicação por metanol, sua relação com a injúria renal aguda (IRA) e as estratégias terapêuticas atualmente utilizadas. O formato de revisão foi selecionado devido à escassez de ensaios clínicos randomizados e à predominância de estudos observacionais e relatos de caso, o que limita a viabilidade de uma meta-análise quantitativa.

O artigo buscou responder à seguinte pergunta: “Quais são os mecanismos fisiopatológicos que explicam a relação entre a intoxicação por metanol e o desenvolvimento de injúria renal aguda, e quais são as abordagens terapêuticas mais eficazes para tratar essa condição?”.

A estratégia de busca utilizou termos relevantes nas bases de dados PubMed, Embase e *Cochrane Library*: (“Methanol” OR “Methyl Alcohol” OR “Methanol Poisoning” OR “Methanol Intoxication” OR “Methanol Toxicity” OR “Methanol Exposure” OR “Methanol-Induced Injury” OR “Toxic Alcohols” OR “Alcohol Poisoning” OR “Alcohol Intoxication”) AND (“Acute Kidney Injury” OR “AKI” OR “Renal Failure” OR “Acute Renal Failure” OR “Acute Renal Injury” OR “Renal Dysfunction” OR “Kidney Failure” OR “Kidney Injury” OR “Kidney Damage” OR “Renal Impairment” OR “Nephrotoxicity” OR “Toxic Nephropathy”) AND (“Pathophysiology” OR “Mechanism of Injury” OR “Mechanism of Toxicity” OR “Mechanism of Action” OR “Pathogenesis” OR “Molecular Mechanism” OR “Mitochondrial Dysfunction” OR “Oxidative Stress” OR “Cellular Toxicity” OR “Metabolic Acidosis”) AND (“Treatment” OR “Therapy” OR “Management” OR “Clinical Management” OR “Supportive Care” OR “Detoxification” OR “Antidote”).

Esta revisão incluiu artigos originais, relatos de caso e séries de casos que abordavam a fisiopatologia da intoxicação por metanol, sua associação com a IRA ou o manejo terapêutico envolvendo antídotos, diálise ou suporte renal. Foram considerados elegíveis estudos em inglês, português ou espanhol; revisados por pares; com texto completo disponível; e publicados entre janeiro de 2000 e outubro de 2025.

Foram excluídos estudos sem texto completo, aqueles focados exclusivamente em etilenoglicol ou outros álcoois sem menção ao metanol, trabalhos com dados insuficientes sobre função renal ou terapêutica, bem como estudos duplicados ou com baixa qualidade metodológica.

A triagem dos estudos ocorreu em duas etapas: avaliação inicial de títulos e resumos para exclusão de estudos irrelevantes, seguida da leitura integral para confirmar elegibilidade. Dois revisores independentes realizaram o processo, resolvendo divergências por consenso. A extração padronizada dos dados incluiu informações sobre autor, ano de publicação, tipo de estudo, características da população/amostra, mecanismos fisiopatológicos, tipo e gravidade da IRA, estratégias terapêuticas (fomepizol, etanol, hemodiálise, ácido folínico, suporte renal) e desfechos renais ou clínicos.

Devido ao desenho narrativo da revisão, a síntese dos dados foi qualitativa e organizada em dois eixos temáticos: (1) fisiopatologia da intoxicação por metanol resultando em IRA, incluindo metabolismo, toxicidade mitocondrial e acidose metabólica; e (2) abordagens terapêuticas e suporte renal, abrangendo antídotos, diálise e correção metabólica.

No total, 574 artigos foram inicialmente identificados; após a remoção de 36 duplicatas, 538 foram avaliados por títulos e resumos, resultando em 17 artigos com texto completo incluídos na análise final.

## RESULTADOS

### Fisiopatologia da Intoxicação por Metanol e Mecanismos de Nefrotoxicidade

A patogênese da injúria renal aguda (IRA) na intoxicação por metanol envolve diversos mecanismos interativos, atuando tanto de forma sistêmica quanto local^4^. Para compreender plenamente suas implicações renais, é essencial, em primeiro lugar, examinar o metabolismo e a fisiopatologia da intoxicação por metanol.

O metanol é rapidamente absorvido pelo trato gastrointestinal, com meia-vida média de cinco minutos e pico de absorção ocorrendo entre trinta e sessenta minutos após a ingestão^1,5,6^. O passo metabólico inicial e mais crítico é a oxidação do metanol a formaldeído, reação catalisada pela enzima hepática álcool desidrogenase (ADH)^3,6,7,8^. O formaldeído, um intermediário altamente reativo, possui meia-vida extremamente curta, de um a dois minutos, antes de ser rapidamente convertido pela enzima aldeído desidrogenase (ALDH) em ácido fórmico (ou formiato)^3,6,7,8^.

Posteriormente, o ácido fórmico se dissocia em íons hidrogênio e no ânion formiato, sendo este último metabolizado pela enzima 10-formiltetraidrofolato desidrogenase. Essa enzima catalisa a combinação do formiato com o tetraidrofolato para formar 10-formiltetraidrofolato, o qual é finalmente degradado a dióxido de carbono e água^5,8^. O tetraidrofolato deriva do ácido fólico, e sua concentração depende da ingestão dietética adequada de folato e da regeneração eficiente durante a oxidação do formiato—um processo fre­quentemente limitado ou saturado em humanos^5,8^.

Como consequência, a meia-vida do ácido fórmico pode se estender por até vinte horas, resultando em acúmulo sustentado de formiato na corrente sanguínea e em forte correlação entre níveis séricos de formiato e toxicidade do metanol^3,5^. Também se propôs que a depuração lenta do ácido fórmico pode refletir uma cinética de ordem zero, afetando tanto o metabolismo do metanol quanto o do ácido fórmico, potencialmente agravada pela reciclagem contínua de ácido fórmico e prótons com íons cloreto no rim^5^.

O acúmulo de ácido fórmico constitui o principal mecanismo subjacente à acentuada acidose metabólica observada na intoxicação por metanol^8^. O ácido fórmico inibe a citocromo c oxidase, bloqueando a fosforilação oxidativa nas mitocôndrias—processo essencial para a respiração celular—e induzindo hipóxia tecidual^5,7,8^. A conse­quente disfunção mitocondrial desencadeia um desvio para o metabolismo anaeróbico, levando à produção excessiva de lactato e agravando a acidose metabólica inicialmente provocada pela dissociação do ácido fórmico^3,5,7^.

O acúmulo combinado de ácido fórmico e ácido láctico, decorrente da utilização celular prejudicada de oxigênio, produz um aumento marcante do ânion gap. A acidose severa resultante agrava a condição, promovendo a difusão intracelular do ácido fórmico^3,5,7^. Em comparação ao formiato, o ácido fórmico não dissociado é aproximadamente três vezes mais potente como inibidor da citocromo c oxidase mitocondrial, a enzima terminal da cadeia respiratória^5^. Assim, tecidos de alta demanda metabólica, baixa reserva energética e abundância mitocondrial—como o nervo óptico e a retina— são particularmente vulneráveis à injúria hipóxica, levando às alterações visuais características da intoxicação por metanol^1,7,8^.

A injúria renal induzida por metabólitos tóxicos, incluindo o ácido fórmico, é mediada por vias universais comuns a diversas formas de IRA. Um desses mecanismos é o estresse oxidativo, resultante da produção excessiva de espécies reativas de oxigênio (ROS), secundárias à disfunção mitocondrial. O rim, especialmente as células dos túbulos proximais, é particularmente suscetível ao dano induzido por ROS. Essa produção exagerada de ROS supera os sistemas antioxidantes intrínsecos, levando à depleção de glutationa reduzida (GSH) e ao acúmulo de subprodutos de peroxidação lipídica (MDA) e de marcadores de oxidação proteica (AOPP, PC)^9,10,11^. Além disso, as mitocôndrias têm papel central na regulação das vias de morte celular, incluindo apoptose, necrose e autofagia, ao ativar processos de morte programada^12^. Ademais, ocorre intensa ativação de vias inflamatórias, particularmente a via do fator nuclear kappa B (NF-κB). Marcadores de estresse celular e inflamação, como a molécula de injúria renal 1 (KIM-1), tornam-se altamente expressos após injúria tubular^9,12^.

Além disso, a ingestão de metanol frequentemente produz um gap osmolar (GO) acentuado, uma vez que uma concentração sérica de metanol de 50 mg/dL corresponde a um aumento aproximado de 17 mOsm/kg de H^2^O^5^. Diante disso, a intoxicação por metanol tem sido associada a uma alteração morfológica específica nos túbulos proximais, semelhante à nefrose osmótica. Esse padrão histopatológico é caracterizado por pinocitose e acúmulo lisossomal de sódio e água, resultando em tumefação celular e distensão vacuolar das células tubulares proximais, sem evidência de comprometimento glomerular^4^, como descrito em duas biópsias renais relatadas por Verhelst^3^.

O influxo de cálcio nas células tubulares foi proposto como um dos possíveis mecanismos de toxicidade. Além disso, a absorção de cloreto de sódio no túbulo proximal é intensamente estimulada pelo formiato, o que pode explicar as alterações hidrópicas observadas nas células epiteliais tubulares proximais nessas biópsias, culminando, por fim, em necrose tubular aguda (NTA)^3,13^. A nefrose osmótica é frequentemente uma forma reversível de injúria tubular proximal inicial, com recuperação funcional típica após a retirada do agente causador, embora casos raros de insuficiência renal persistente tenham sido descritos^4^.

Em alguns casos, o rim não é considerado alvo direto da toxicidade pelo metanol; em vez disso, a IRA é vista como uma complicação secundária à progressão da doença, decorrente de mecanismos indiretos de injúria^3^. A hemólise é considerada um desses mecanismos. Sugeriu-se que o ácido fórmico pode induzir hemólise por um efeito citotóxico sobre os eritrócitos—um processo mais relacionado ao grau de acidose metabólica do que a uma ação hemolítica direta sobre as hemácias^3^. Proteínas heme podem promover injúria renal por três mecanismos principais: redução da perfusão renal, citotoxicidade direta e formação de cilindros pigmentares, levando à obstrução tubular^1^.

A rabdomiólise não é uma complicação comum da intoxicação por metanol; entretanto, também foi identificada como um fator indireto de risco para IRA^1^. O mecanismo envolve a precipitação de mioglobina e hemoglobina nos túbulos renais, causando obstrução tubular. Essa precipitação é exacerbada pelo pH urinário ácido^6,7^. Em um dos estudos analisados, 7 dos 11 casos de IRA apresentaram mioglobinúria e hemoglobinúria^3^. Níveis elevados de creatinoquinase (CPK) foram relatados em surtos epidêmicos de intoxicação por metanol, sugerindo uma possível associação com a rabdomiólise^13^. No entanto, nenhum efeito tóxico direto do metanol sobre o músculo esquelético foi claramente demonstrado na literatura, nem propriedades hemolíticas diretas foram atribuídas a ele^3^.

Por fim, a pancreatite aguda, uma complicação comum nos casos graves de intoxicação, pode contribuir para a IRA por meio da liberação sistêmica de mediadores inflamatórios e enzimas pancreáticas provenientes de tecido necrótico, resultando em nefrotoxicidade direta^7^, como mostrado na Figura 1.

**Figura 1:** Fisiopatologia da intoxicação por metanol e mecanismos de nefrotoxicidade.

Abreviaturas – ATP: Trifosfato de adenosina; NF-κB: Fator nuclear kappa B; TNF-a: Fator de necrose tumoral alfa; IL-1: Interleucina 1; IL-6: Interleucina 6.

Notas – O metanol é metabolizado em ácido fórmico, causando disfunção mitocondrial, estresse oxidativo, depleção de ATP e morte celular. No rim, a injúria do túbulo proximal resulta em nefrose osmótica, com vacuolização citoplasmática, ativação do NF-κB, liberação de citocinas pró-inflamatórias, recrutamento de macrófagos e progressão para necrose tubular aguda.

### Abordagens Terapêuticas

O manejo da intoxicação por metanol, incluindo, de forma crítica, a abordagem da injúria renal aguda (IRA) associada, baseia-se em diversas intervenções fundamentais: interromper a produção de metabólitos tóxicos, facilitar a eliminação tanto do metanol quanto do ácido fórmico, corrigir a acidose metabólica potencialmente fatal e fornecer suporte clínico abrangente^8^.

### Etanol como Inibidor da ADH

O etanol pode ser utilizado como antídoto alternativo quando o fomepizol não está disponível, atuando como substrato competitivo da enzima álcool desidrogenase (ADH), com afinidade aproximadamente 10 a 20 vezes maior que a do metanol. O objetivo terapêutico é manter uma concentração sérica de etanol em torno de 100 mg/dL (22 mmol/L), suficiente para saturar a ADH e impedir o metabolismo do metanol em formaldeído e ácido fórmico^7,8,12,14^.

O etanol intravenoso é geralmente administrado como solução a 10%. Uma dose de ataque de 0,6–0,8 g/kg é infundida ao longo de 30–60 minutos, seguida por infusão de manutenção de 66–154 mg/kg/h, ajustada conforme idade, uso crônico de álcool e níveis séricos de etanol. Durante a hemodiálise, a depuração do etanol é significativa­mente aumentada, e a taxa de infusão deve ser elevada em 2 a 3 vezes para manter concentrações terapêuticas séricas^7,8,12,14^.

Quando o etanol intravenoso não está disponível, pode-se utilizar a administração oral ou por sonda nasogástrica. Administra-se uma dose de ataque equivalente a 0,8 g/kg, seguida de 0,1–0,2 g/kg/h, utilizando etanol diluído (por exemplo, destilados diluídos a uma concentração conhecida). É necessário o monitoramento frequente dos níveis séricos de etanol para garantir inibição adequada da ADH e evitar toxicidade^7,8,12,14^.

### Inibição do Metabolismo do Metanol: Bloqueio da ADH

Prevenir a conversão do metanol em formaldeído e, subsequentemente, em ácido fórmico é um objetivo terapêutico central. Isso é alcançado por meio da inibição da ADH, a enzima limitante dessa via metabólica^8^. Os dois inibidores de ADH disponíveis são o fomepizol e o etanol.

**Fomepizol (4-metilpirazol):** O fomepizol apresenta afinidade marcadamente maior pela ADH (estimada em 500 a 1000 vezes superior à do etanol), permitindo inibição completa da enzima em concentrações séricas relativamente baixas (alvo >0,8 mg/L ou 10 µmol/L)^8^. Evidências clínicas provenientes de estudos observacionais demonstram sua eficácia na redução dos níveis de ácido fórmico, na melhora da acidose metabólica e, potencialmente, na melhora dos desfechos visuais na intoxicação por metanol^8,15^. Entre suas vantagens estão o perfil favorável de efeitos adversos (relatos de cefaleia, náusea e tontura), farmacocinética mais previsível, com menor necessidade de monitorização intensiva de níveis séricos, ausência de efeitos sedativos (ao contrário do etanol) e ausência de contribuição para o gap osmolar sérico, o que pode ser útil para o acompanhamento indireto da eliminação do metanol quando níveis diretos não estão disponíveis^8,15^. Dessa forma, constitui o agente de escolha quando acessível. Suas principais limitações são o alto custo e a disponibilidade variável entre os serviços de saúde. Como o fomepizol é removido pela hemodiálise, ajustes de dose (aumento da frequência ou doses suplementares) são necessários durante a terapia de substituição renal. O esquema padrão envolve uma dose de ataque intravenosa de 15 mg/kg, seguida por doses de manutenção programadas (por exemplo, 10 mg/kg a cada 12 horas por 4 doses, depois 15 mg/kg a cada 12 horas), com aumento da frequência durante a diálise^8^. O fomepizol (também conhecido como 4-MP) também inibe o CYP2E1, enzima envolvida no metabolismo do paracetamol, o que confere proteção contra IRA induzida por paracetamol em modelos murinos^16^, embora a inibição da ADH permaneça o principal mecanismo terapêutico citado na intoxicação por metanol.

**Etanol:** O etanol atua como substrato competitivo da ADH, com afinidade 10 a 20 vezes maior que a do metanol^8^. Para inibição eficaz da enzima e do metabolismo do metanol, é necessário alcançar e manter uma concentração sérica de etanol em torno de 100 mg/dL^7,8^. As principais vantagens do etanol são o baixo custo e a ampla disponibilidade. Entretanto, ele apresenta desvantagens significativas, incluindo depressão do sistema nervoso central (dificultando a avaliação neurológica), necessidade de monitorização frequente dos níveis séricos e ajustes de dose (geralmente exigindo internação em UTI), aumento da osmolalidade sérica (comprometendo o uso do gap osmolar como ferramenta de monitoramento), risco de hipoglicemia, potenciais efeitos hepáticos e remoção substancial pela hemodiálise, exigindo aumento expressivo das taxas de infusão durante o procedimento. Apesar dessas limitações, o etanol permanece uma alternativa viável quando o fomepizol não pode ser utilizado. A posologia envolve uma dose de ataque seguida por infusão de manutenção, que deve ser significativamente aumentada durante a hemodiálise^8^.

### Indicações para Bloqueio da ADH

Os critérios de diretrizes para iniciar o bloqueio da ADH (com fomepizol ou etanol), conforme citado por Kraut e Kurtz (2008), incluem: níveis plasmáticos confirmados de metanol >20 mg/dL; história documentada de ingestão recente associada a gap osmolar >10 mOsm/L; ou forte suspeita clínica baseada na apresentação (por exemplo, sintomas visuais, acidose), combinada a achados laboratoriais compatíveis (pH arterial <7,3, bicarbonato sérico <20 mEq/L ou gap osmolar >20 mOsm/L). A administração precoce é funda­mental para a eficácia, enquanto ainda há metanol não metabolizado em circulação^8,15^.

### Remoção Extracorpórea: Hemodiálise

A hemodiálise (HD) é uma intervenção crucial nos casos graves de intoxicação por metanol, com o objetivo principal de acelerar a remoção tanto do metanol quanto de seu metabólito tóxico, o ácido fórmico, da circulação^7,8^, conforme ilustrado na Figura 2. As propriedades físico-químicas dessas substâncias (baixo peso molecular, mínima ligação a proteínas e baixo volume de distribuição) tornam-nas altamente passíveis de remoção por diálise^8,17^. A hemodiálise intermitente proporciona depuração mais rápida quando comparada a outras modalidades de terapia renal substitutiva, como terapias contínuas ou diálise peritoneal^8^.

**Figura 2:** Fluxograma da abordagem diagnóstica e terapêutica da intoxicação por metanol, incluindo critérios para hemodiálise.

#### Indicações para hemodiálise

A decisão de iniciar HD baseia-se em parâmetros clínicos e laboratoriais indicativos de intoxicação grave. As indicações comumente aceitas incluem:

- **Níveis séricos elevados de metanol:** os limiares variam, sendo frequentemente citados valores >50 mg/dL ou, em alguns contextos, >25 mg/dL^7,8^.
- **Acidose metabólica grave:** definida por parâmetros como pH arterial <7,1, <7,25 ou <7,30, frequentemente refratária à terapia isolada com bicarbonato^7,8^. Bicarbonato sérico baixo, por si só, é um forte preditor de mortalidade e pode sustentar implicitamente a necessidade de diálise^7^.
- **Comprometimento visual:** qualquer grau de distúrbio visual atribuído ao metanol é frequentemente considerado indicação^7,8^. A presença de cegueira na admissão aumenta significativamente o risco de mortalidade^7^.
- **Presença de IRA / insuficiência renal:** a injúria renal constitui, por si só, uma indicação para terapia renal substitutiva, especialmente quando grave ou quando contribui para acidose e distúrbios eletrolíticos^7,8^.
- **Deterioração clínica:** incluindo piora dos sinais vitais (por exemplo, hipotensão persistente) ou distúrbios eletrolíticos significativos (como hipercalemia grave, embora geralmente menos comum que a acidose), não manejáveis por outros meios^7,8^.

Embora o papel primário da HD seja a remoção do metanol, ela também remove eficazmente o formiato. Estudos demonstram que a HD pode reduzir substancialmente a meia-vida do formiato, especialmente em casos graves, nos quais a depuração endógena pode estar sobrecarregada. Além disso, a HD corrige de forma eficiente a acidose metabólica por meio da transferência de bicarbonato a partir do dialisato^8^.

##### Momento e duração

Recomenda-se o início precoce da HD, idealmente de forma concomitante ao bloqueio da ADH (preferencialmente com fomepizol), na maioria dos pacientes com intoxicação significativa por metanol, a fim de acelerar a depuração da toxina, manejar a acidose, melhorar os desfechos e potencialmente mitigar a gravidade ou o desenvolvimento de IRA^8,15^. Na prática, em cenários de surtos, a iniciação dentro de 3–5 horas após a chegada ao pronto-socorro tem sido alcançada^15^. A duração da HD é orientada pelo objetivo de reduzir os níveis de metanol a concentrações não tóxicas (por exemplo, <16 mg/dL ou <5 mmol/L, ou indetectáveis) e de resolver a acidose metabólica (pH >7,3). Fórmulas cinéticas que incorporam parâmetros do paciente e características do dialisador podem auxiliar na estimativa do tempo necessário de diálise^8^. Os pacientes podem necessitar de múltiplas sessões de HD (uma a três ou mais), dependendo da carga inicial de metanol, da metabolização contínua, caso o bloqueio da ADH seja incompleto, e da gravidade da intoxicação^15^. O monitoramento dos níveis de metanol e do estado ácido-base é essencial para determinar o ponto final da terapia.

#### Correção da Acidose Metabólica: Bicarbonato de Sódio

A administração intravenosa de bicarbonato de sódio é uma medida de suporte vital para contrabalançar a acidose metabólica grave causada pelo acúmulo de ácido fórmico^7,8,15^. A melhora da acidemia pode otimizar a função cardiovascular (ao aumentar a contratilidade miocárdica e a responsividade vascular às catecolaminas) e, teoricamente, reduzir a toxicidade do ácido fórmico ao diminuir sua fração não ionizada, limitando assim sua penetração tecidual (especialmente no SNC e no nervo óptico) e possivelmente aumentando sua excreção urinária por aprisionamento iônico. A terapia com bicarbonato é geralmente indicada quando o pH arterial cai abaixo de 7,3^7,8^, sendo frequentemente necessária uma correção mais agressiva em casos de acidemia grave (pH <7,1–7,2)^8^. Pode-se administrar uma dose inicial em bolus de 1–2 mEq/kg, seguida de infusão contínua ou bolus intermitentes, titulados com base em gasometrias arteriais frequentes para atingir um pH-alvo (por exemplo, >7,3)^7^. É fundamental evitar a correção excessiva e monitorar potenciais complicações, como hipocalemia ou hipocalcemia. A hemodiálise permanece o método mais eficaz para corrigir a acidose grave, devido à sua capacidade de fornecer grandes quantidades de tampão bicarbonato^8^.

#### Manejo de Suporte

O cuidado intensivo de suporte, adaptado ao estado clínico do paciente, é essencial em paralelo aos tratamentos específicos. O manejo das vias aéreas, incluindo intubação orotraqueal e ventilação mecânica, é frequentemente necessário em pacientes que se apresentam com rebaixamento importante do nível de consciência (escores baixos na Escala de Coma de Glasgow), convulsões ou comprometi­mento respiratório decorrente de acidose grave ou depressão do SNC^7,15^. O suporte cardiovascular é crítico; a hipotensão, marcador de toxicidade grave e mau prognóstico, requer manejo agressivo com reposição volêmica intravenosa e, frequentemente, uso de vasopressores ou inotrópicos (por exemplo, noradrenalina) para manter a pressão arterial média (PAM) adequada e a perfusão orgânica, incluindo a perfusão renal^7,8^. Métodos de descontaminação gastrointestinal, como lavagem gástrica ou carvão ativado, são geralmente considerados ineficazes, exceto quando realizados dentro de uma janela muito curta (30–60 minutos) após a ingestão, devido à rápida absorção do metanol^8^. O monitoramento contínuo e vigilante dos sinais vitais (frequência cardíaca, pressão arterial, frequência respiratória, temperatura), do estado neurológico (ECG/Glasgow), do equilíbrio ácido-base (gasometrias seriadas), dos eletrólitos (especialmente potássio), da glicemia, da função renal (creatinina, ureia, débito urinário) e da acuidade visual é obrigatório durante todo o tratamento^7,8^. As convulsões, quando presentes, devem ser tratadas com benzodiazepínicos conforme os protocolos padrão^15^.

## DISCUSSÃO

### Considerações Prognósticas e Importância do Tempo de Intervenção

O desfecho clínico após a intoxicação por metanol, incluindo o desenvolvimento e a gravidade da injúria renal aguda (IRA), depende criticamente da intensidade da acidose metabólica na apresentação e, sobretudo, do intervalo de tempo entre a ingestão e o início de terapias eficazes, como o bloqueio da ADH e a hemodiálise. Fatores fortemente preditivos de mortalidade e morbidade significativa (incluindo IRA e perda visual permanente) incluem acidemia grave (pH < 7,1, bicarbonato marcadamente reduzido, ânion gap elevado, lactato elevado), coma profundo (baixo escore na Escala de Coma de Glasgow), instabilidade hemodinâmica (hipotensão, baixa pressão arterial média), presença de cegueira na admissão e evidência de IRA (elevação da creatinina sérica)^7,8^. Atrasos na procura por atendimento médico, influenciados por fatores como falta de conscientização ou medo de repercussões legais em determinadas regiões, aumentam significativamente o risco de desfechos adversos, especialmente cegueira irreversível^7^. Em contrapartida, séries de casos e relatos de surtos sugerem que o diagnóstico rápido associado à iniciação precoce de terapia combinada—bloqueioda ADH (preferencialmente com fomepizol) e hemodiálise precoce e eficaz—de resultar em taxas de mortalidade substancialmente menores e melhor recuperação funcional quando comparado a cenários com atraso terapêutico ou manejo menos agressivo^15^. Assim, minimizar o tempo até o tratamento é fundamental para limitar o acúmulo de ácido fórmico e prevenir ou atenuar danos irreversíveis aos rins, aos olhos e ao sistema nervoso central^7,15^.

### Fatores de Risco para IRA e Prognóstico

A IRA é frequentemente agravada por complicações sistêmicas da intoxicação grave, notadamente acidose metabólica profunda e hipotensão, refletidas por redução da pressão arterial média (PAM)^7,14^. Essa instabilidade hemodinâmica compromete a perfusão renal e potencializa os efeitos tóxicos do formiato. Além disso, pacientes com doença renal crônica (DRC) pré-existente ou função renal comprometida apresentam risco ainda maior de nefrotoxicidade relacionada ao metanol, particularmente o desenvolvimento de nefrose osmótica, devido à menor capacidade de metabolizar e excretar o composto tóxico^4^.

No estudo de Thongprayoon^1^, em uma coorte de 603 pacientes hospitalizados com diagnóstico primário de intoxicação por metanol, 135 desenvolveram IRA na admissão. A análise multivariada identificou anemia (OR 3,43; IC 95% 3,43–6,62; p < 0,001), hipertensão arterial (OR 1,86; IC 95% 1,10–3,15; p = 0,02), depleção volêmica (OR 3,46; IC 95% 1,70–7,06; p = 0,001), sepse (OR 6,91; IC 95% 2,38–20,05; p < 0,001), rabdomiólise (OR 6,25; IC 95% 1,84–21,26; p = 0,003) e pancreatite aguda (OR 5,30; IC 95% 1,71–16,44; p = 0,004) como fatores de risco independentes para o desenvolvimento de IRA em pacientes com intoxicação por metanol^1^. Entre esses, a sepse apresentou a associação mais forte com IRA, possivelmente explicada pela hipoperfusão renal decorrente da instabilidade hemodinâmica, disfunção microcirculatória renal, resposta inflamatória sistêmica, levando à injúria tubular celular—predominantemente necrose tubular aguda—e subsequente disfunção mito­condrial, ainda mais agravada pela cito­toxicidade intrínseca do ácido fórmico^1,7^.

Adicionalmente, demonstrou-se que pacientes com IRA apresentaram necessidade significativamente maior de ventilação mecânica invasiva (OR 6,93; IC 95% 4,08–11,70; p < 0,001) e de terapias extracorpóreas (OR 4,06; IC 95% 2,58–6,38; p < 0,001), em comparação àqueles sem IRA. Ademais, a IRA esteve significativamente associada a maior risco de falência respiratória (OR 7,53; IC 95% 4,43–12,80; p < 0,001), circulatória (OR 3,15; IC 95% 1,41–7,04; p = 0,005), neurológica (OR 2,66; IC 95% 1,55–4,57; p < 0,001) e hema­tológica (OR 2,90; IC 95% 1,25–6,77; p = 0,01). Notavelmente, a presença de IRA conferiu uma chance 6,39 vezes maior de mortalidade intra-hospitalar em comparação à dos pacientes sem IRA (OR 6,39; IC 95% 2,92–13,96; p < 0,001). Esses achados podem ser explicados pelas consequências sistêmicas da disfunção renal. A IRA leva à retenção de sal e água, acompanhada de redução da contratilidade miocárdica e arritmias cardíacas—ambas desencadeadas por distúrbios eletrolíticos e do equilíbrio ácido-base—que, em conjunto, contribuem para o desenvolvimento de edema pulmonar hidrostático. Além disso, a inflamação sistêmica e a uremia podem aumentar a per­meabilidade vascular pulmonar, resultando em edema pulmonar não hidrostático, outro mecanismo de insuficiência respiratória^1^.

Esses mecanismos são consistentes com o modelo geral de IRA induzida por intoxicação, que enfatiza a convergência de estresse oxidativo, injúria mitocondrial e inflamação como mediadores centrais do dano renal tóxico em diversas etiologias. O metanol se insere nesse paradigma ao provocar falência mitocondrial e ativação de vias inflamatórias, particularmente por meio da via do NF-κB e da superexpressão de marcadores de injúria renal, como o KIM-1. A combinação de disfunção mitocondrial e estresse inflamatório explica a acidose grave, a apoptose tubular e a recuperação renal tardia observadas em muitos casos^9,17,18,19^.

Sob uma perspectiva comparativa, a nefropatia induzida por metanol compartilha algumas semelhanças patogênicas com a intoxicação por etilenoglicol, outra causa de injúria renal tóxica aguda. Contudo, o etilenoglicol provoca principal­mente obstrução tubular induzida por cristais, devido à deposição de oxalato de cálcio, enquanto a toxicidade do metanol leva predominantemente à disfunção metabólica e mitocondrial. Apesar dessas diferenças mecanísticas, ambas as condições frequentemente culminam em necrose tubular aguda e disfunção renal clinicamente significativa. Na intoxicação por metanol, a ausência de deposição cristalina ressalta um perfil fisiopatológico distinto, impulsionado por inibição bioquímica, em vez de obstrução mecânica, reforçando o papel central da IRA como marcador de gravidade da doença e determinante-chave da tomada de decisão terapêutica, particularmente quanto à indicação precoce de terapia renal substitutiva^4,19,20^.

Além da toxicidade tubular direta, diversos fatores secundários agravam ainda mais a injúria renal e influenciam diretamente o manejo nefrológico. Instabilidade hemodinâmica, desidratação e hipotensão comprometem a perfusão renal e promovem dano tubular isquêmico, frequentemente exigindo otimização hemodinâmica agressiva e suporte renal precoce^1^. Adicionalmente, rabdomiólise e hemólise, embora menos frequentes, podem contribuir para nefropatia por pigmentos, por meio da liberação de mioglobina e hemoglobina, especialmente em ambientes urinários ácidos, reduzindo o limiar para o início da terapia renal substitutiva^3,8^. A sepse, identificada como fator de risco independente, intensifica a inflamação sistêmica e a disfunção mitocondrial, criando um ciclo autoalimentado de injúria renal e disfunção multiorgânica. Nesse contexto, a presença e a gravidade da IRA emergem não apenas como complicações, mas como determinantes centrais do prognóstico e da estratégia terapêutica, ressaltando o papel crítico do nefrologista na indicação oportuna da diálise e no suporte renal abrangente^1,20^.

## CONCLUSÃO

A intoxicação por metanol é uma emergência médica caracterizada por toxicidade sistêmica complexa. Entre suas complicações, a injúria renal aguda destaca-se tanto como marcador de gravidade da doença quanto como determinante prognóstico. O dano renal é multifatorial, envolvendo toxicidade tubular direta pelo ácido fórmico, estresse oxidativo, disfunção mitocondrial e instabilidade hemodinâmica. A intervenção precoce com fomepizol, ácido folínico e hemodiálise, associada a suporte clínico rigoroso, reduz significativamente a mortalidade e melhora os desfechos renais. A compreensão da fisiopatologia renal na intoxicação por metanol é essencial para orientar decisões clínicas e para o desenvolvimento de novas estratégias tera­pêuticas voltadas à proteção e à recuperação renal.

## Data Availability

O conjunto de dados que fundamenta os resultados deste estudo não está disponível publicamente.
